# Therapeutic and prognostic potential of GPCRs in prostate cancer from multi-omics landscape

**DOI:** 10.3389/fphar.2022.997664

**Published:** 2022-08-30

**Authors:** Shiqi Li, Jianfang Chen, Xin Chen, Jin Yu, Yanzhi Guo, Menglong Li, Xuemei Pu

**Affiliations:** ^1^ College of Chemistry, Sichuan University, Chengdu, China; ^2^ Department of Physics and Astronomy, University of California, Irvine, Irvine, CA, United States

**Keywords:** G protein-coupled receptors, prostate cancer, multi-omics, virtual screening, prognostic model

## Abstract

Prostate cancer (PRAD) is a common and fatal malignancy. It is difficult to manage clinically due to drug resistance and poor prognosis, thus creating an urgent need for novel therapeutic targets and prognostic biomarkers. Although G protein-coupled receptors (GPCRs) have been most attractive for drug development, there have been lack of an exhaustive assessment on GPCRs in PRAD like their molecular features, prognostic and therapeutic values. To close this gap, we herein systematically investigate multi-omics profiling for GPCRs in the primary PRAD by analyzing somatic mutations, somatic copy-number alterations (SCNAs), DNA methylation and mRNA expression. GPCRs exhibit low expression levels and mutation frequencies while SCNAs are more prevalent. 46 and 255 disease-related GPCRs are identified by the mRNA expression and DNA methylation analysis, respectively, complementing information lack in the genome analysis. In addition, the genomic alterations do not exhibit an observable correlation with the GPCR expression, reflecting the complex regulatory processes from DNA to RNA. Conversely, a tight association is observed between the DNA methylation and mRNA expression. The virtual screening and molecular dynamics simulation further identify four potential drugs in repositioning to PRAD. The combination of 3 clinical characteristics and 26 GPCR molecular features revealed by the transcriptome and genome exhibit good performance in predicting progression-free survival in patients with the primary PRAD, providing candidates as new biomarkers. These observations from the multi-omics analysis on GPCRs provide new insights into the underlying mechanism of primary PRAD and potential of GPCRs in developing therapeutic strategies on PRAD.

## Introduction

Prostate cancer (PRAD) is the second commonly diagnosed cancer and leading cause of mortality in men worldwide. 248,530 new cases were reported in 2021, resulting in approximately 3.4 thousand deaths (Siegel et al., 2021). With the popularity of early prostate specific antigen (PSA) screening, increasing cases are being detected (Force, 2018). When PRAD is diagnosed, difficult clinical decision is generally faced by both clinicians and patients, as it is very difficult to predict whether a patient will progress to aggressive and metastatic disease. Currently, screening, diagnosis and prognosis of PRAD are heavily dependent on clinical characteristics like age, tumor stage and Gleason score, while these indicators cannot well distinguish individuals with different survival outcomes at the beginning of the disease, leading to either over- or under-treatment of these patients (Cooperberg et al., 2010; Klotz et al., 2014). Therefore, it is highly desired to find new biomarkers to complement existing clinical tools for diagnostic, prognostic and disease monitoring such that improve risk stratification of the PRAD patients and develop effective and precise therapeutic targets. A global analysis of multi-omics data provides a potential solution for gaining insight into the underlying mechanisms of disease development, which can better stratify patients and uncovers new therapeutic targets.

G protein-coupled receptors (GPCRs), with a seven-transmembrane domain structure, an extracellular amino terminus and an intracellular carboxyl terminus, are the largest and most diverse protein family of cell surface receptors in many species, accounting for ∼4% of the encoded human genome (Pierce et al., 2002; Fredriksson et al., 2003). As key transducers of signals from the extracellular milieu to the inside of the cell, GPCRs can regulate a vast array of fundamental biological processes, including cellular growth and metabolism, hormone regulation, sensory perception, and alterations in neuronal activity. Consequently, their aberrant activity or expression also leads to many common diseases and disorders, for example, diabetes, Alzheimer disease, depression, and heart failure (Hauser et al., 2017; Raimondi et al., 2019). The critical roles of GPCRs in numerous physiological functions drive them to be the largest family of drug targets (Hauser et al., 2017). However, this family has not typically been a major focus for oncology drug discovery and only a handful of GPCRs were approved to be targets of anti-cancer drugs (Innamorati et al., 2011; Nieto Gutierrez and McDonald, 2018; Wu et al., 2019), although their oncogenic potential has been known for more than 30 years. The first study linking a GPCR to tumorigenic activity was reported by Young et al. (1986). Recently, increasing evidences indicated that GPCRs can contribute to many facets of tumorigenesis, including angiogenesis, immune evasion, growth, and apoptosis of tumor cells (Nieto Gutierrez and McDonald, 2018; Wang et al., 2019; Wu et al., 2019; Kaushik et al., 2020). In addition, functional roles of certain GPCR members in cancers are gradually being appreciated. For example, GPR30 is overexpressed in a variety of malignances including PRAD (Siegfried et al., 2009; Chan et al., 2010; Chevalier et al., 2012), and its overexpression has been reported to be involved in lower survival rates in patients with endometrial or ovarian cancer, and an elevated risk of developing metastases in patients with breast cancer (Filardo et al., 2006; Smith et al., 2007; Albanito et al., 2008; Pandey et al., 2009; Du et al., 2012; Zhu et al., 2017). The knockdown of GPRC6A in PC3 cells was reported to significantly decrease tumor metastatic efficiency and invasiveness while increased expression of GPRC6A would enhance ERK and EMT signaling (Liu et al., 2016). Abnormalities in Formylpeptide receptor-2 could induce autonomous migration and proliferation of colon cancer cells (Xiang et al., 2016). GPR18 was revealed to involve in inhibiting apoptosis and increasing the survival rate of melanoma cells (Qin et al., 2011). Activation of S1P2R could mediate inhibition of glioblastoma cell viability (Malchinkhuu et al., 2008). Malignant cells use chemokine receptors to migrate to distant sites of ligand expression, for example, CCR7 and CCR10 were demonstrated to participate in metastatic homing of cancer cells and cell growth (Balkwill, 2004; O'Hayre et al., 2008). These findings clearly show potential of GPCR as novel targets in the cancer treatment. Thus, targeting GPCRs to mediate signaling pathways is currently considered as a promising strategy for drug discovery of the cancers (Sriram and Insel, 2018; Chaudhary and Kim, 2021).

Although the correlation of GPCRs with oncology has been confirmed by growing studies, previous works mainly focused on a few members of GPCRs. Consequently, some receptors were well studied due to their well-known importance while others were ignored. In addition, Insel P.A. et al. indicated that many GPCRs are not well understood and findings derived from specific GPCRs may not be applicable to all, or even most GPCRs (Insel et al., 2019). Unfortunately, it has been lacked of an unbiased and comprehensive study on the molecular characteristics, prognostic potential, and biological functions for the entire GPCR family in PRAD. To close this gap, we analyze multi-omics data from gene expression, somatic mutation, somatic copy-number alterations (SCNAs) and DNA methylation of patients with primary PRAD to 1) provide a global landscape of aberrations in GPCRs at genomic, epigenetic, and transcriptomic levels; 2) probe impact of upstream features on the mRNA expression; 3) identify receptors that may be served as potential targets for primary PRAD therapy in order to find some drug candidates by molecular dynamics simulation and virtual screening; 4) develop a prognostic model for primary PRAD based on the features derived from the multi-omics analysis. Collectively, we provide the first comprehensive multi-omics analysis for GPCRs in the primary PRAD, which offer insights into therapeutic targets and prognostic value of the GPCR family.

## Results and discussion

### mRNA expression of GPCRs in primary PRAD

As accepted, disease-related genes could be identified by comparing the expression level of genes in normal and tumor tissues (Maiga et al., 2016; Insel et al., 2019; Sriram et al., 2019). Thus, in order to provide a comprehensive evaluation of GPCRs expression in primary PRAD, we first integrated RNA-seq data of 767 GPCR members in normal and tumor tissues using TOIL GTEx and TCGA RNA-seq datasets from UCSC Xena (Goldman et al., 2020). Supplementary Table S1 lists the detailed information about the 767 GPCRs. It can be seen that 228 GPCRs are highly expressed, judged from their median expressions in tumor samples≥10 TPM. However, most of the family (n = 461, 60.10%) are expressed at extremely low levels, which are barely detectable in PRAD tumor samples due to their median expressions less than 1 TPM.

**FIGURE 1:**
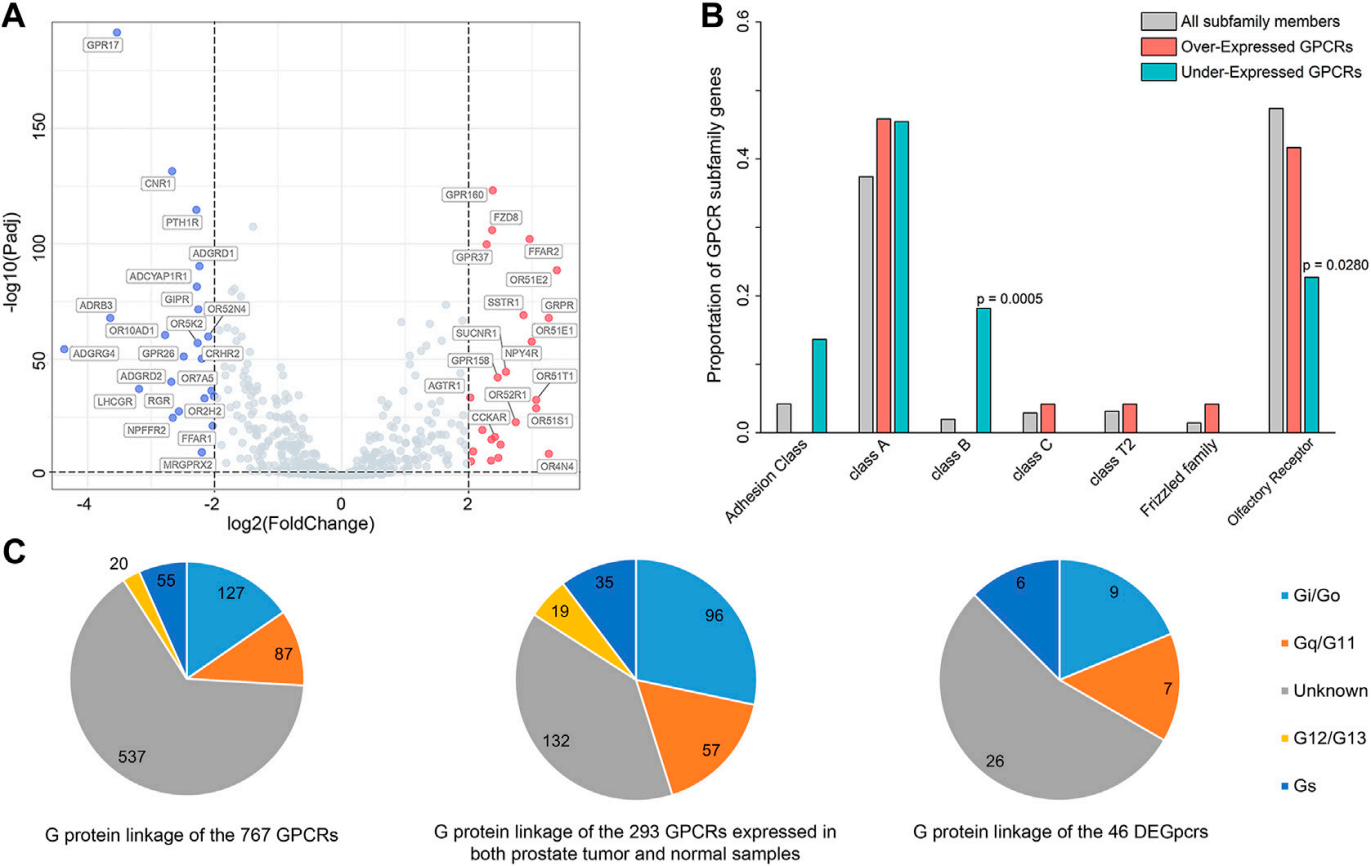
Differentially expressed GPCRs (DEGpcrs) between 495 primary prostate tumor samples and 151 normal samples. **(A)** Volcano plot showing the DEGpcrs. Red and blue dots represent the significantly up- and downregulated genes, respectively. **(B)** Barplot showing subfamily distribution of the DEGpcrs. **(C)** G protein linkage of all the 767 GPCRs (right), the 293 GPCRs expressed in both prostate tumor and normal samples (middle), and the 46 DEGpcrs (left).

We further extracted mRNA-seq data from 495 primary prostate tumor samples and 151 normal samples to do a comparison, through which a total of 46 differentially expressed GPCRs (DEGpcrs, FDR <0.05 and |log2 Fold Change| > 2) were identified. 24 and 22 genes are significantly over- and under-expressed in primary prostate tumors, respectively (vide Supplementary Table S2 and Figure 1A). The aberrant expression of certain DEGpcrs and their involvement in cancer were reported in previous works. For example, *OR51E1*, *OR51E2* and *GPR160*, which are highly expressed (>10 TPM) and significantly upregulated in our analysis, were reported to have antitumor potential for PRAD in previous studies. Specifically, *OR51E1* and *OR51E2* were reported to suppress proliferation and promote cell death in LNCaP cells (Xu et al., 2000; Weng et al., 2005; Rodriguez et al., 2014; Pronin and Slepak, 2021), thus being considered as prostate-specific GPCRs. A much higher level of *GPR160* expression was observed in human prostate cancer cells than that seen in normal prostate tissue and cells, and its knockdown was found to induce apoptosis and cell cycle arrest (Zhou et al., 2016; Guo et al., 2021). Moreover, it is noteworthy that downregulated *LHCGR* is already targeted by Goserelin and Buserelin for the treatment of prostate and breast cancer, and its transcriptional mis-regulation was reported to be closely related with other solid tumors (Tomera et al., 2001; Kirby et al., 2009; Doroszko et al., 2019; Zhong et al., 2019; Lorenzen et al., 2020). In addition, 11 of the 46 DEGpcrs identified are served as targets of approved drugs, e.g., *AGTR1* is commonly used as a target for hypertension drugs like Valsartan, Olmesartan and Losartan (Siragy et al., 2002; Warner and Jarvis, 2002; Azizi et al., 2004). *ADRB3* is a target for the asthma treatment Salmeterol (Hoffmann et al., 2004), and *PTH1R* is targeted by the osteoporosis drugs like Teriparatide and Abaloparatide (Hattersley et al., 2016; Jolette et al., 2017). Combining our findings from PRAD, it is reasonable to assume that these drugs have the potential to be repurposed for treatment of oncology.

Next, we analyzed the distribution of DEGpcrs in different receptor subfamilies and the results are shown in Figure 1B, also seeing Supplementary Table S1 for more details. GPCRs with significant over-expression in PRAD come from five subfamilies while the under-expressed receptors are mainly distributed across four subfamilies. In brief, Class A and Olfactory Receptors include both over- and under-expressed GPCRs, whereas Class D and Other 7TM proteins do not express differentially between the tumor and normal tissues. Then, we used a Fisher’s exact test to do a class enrichment analysis and the result shows that the under-expressed GPCRs in primary PRAD are significantly enriched in Class B (Fisher’s exact test, *p* = 0.0005) and Olfactory Receptor (Fisher’s exact test, *p* = 0.0280), while the other subfamilies do not exhibit significant enrichment (Figure 1B). These observations indicate that certain GPCR subfamilies (e.g., Class B and Olfactory Receptor) are more prone to be dysregulated in PRAD.

To gain functional insights into the dysregulated GPCRs, we first performed a Pearson correlation analysis based on the expression data from 495 primary PRAD patients in TCGA. The result shows that 109 other protein-coding genes present significant and positive correlations with the expression of 5 DEGpcrs (namely, *FAD8*, *GPRP*, *GPR160*, and *NPY4R*, Supplementary Table S3). Then, Metascape (Zhou et al., 2019), a free gene annotation web tool, was employed to conduct pathway enrichment analysis of the 114 genes. As reflected by Supplementary Figure S1A, they are significantly enriched in some cancer-related biological processes, including DNA repair, VEGFA-VEGFR2 signaling pathway, Global Genome Nucleotide Excision Repair (GG-NER), and transcriptional misregulation. Notably, two entries significantly enriched in DisGeNET (low grade prostatic intraepithelial neoplasia and prostatic intraepithelial neoplasia) both exhibit correlations with PRAD, as evidenced by Supplementary Figure S1B. Taken together, the 5 DEGpcrs and their co-expressed genes are indeed involved in cancer-related biological processes, implying their important roles in the PRAD development and progression.

In addition, to in depth comprehend the GPCR role in physiology and disease, we further grouped GPCRs in terms of their coupling preferences to different types of G-proteins (Supplementary Table S1), as it is well-known that the involvement of G proteins as intermediate transducers plays a critical role in GPCR signaling (Southan et al., 2016). As shown in Figure 1C, of the 293 GPCRs expressed in both prostate tumor and normal samples (with median expression in tumors >0.1 TPM), most (132 GPCRs) have unknown G-protein linkages. More GPCRs in the rest couple to G_i/o_ (96 GPCRs) with respect to the other G proteins, followed by G_q/11_ (57 GPCRs) (Figure 1C). This observation is in line with a previous pan-cancer analysis that GPCRs expressed in both tissues and tumors most frequently couple to G_i/o_ and G_q/11_ (Sriram et al., 2019). Also, the G_i/o_-GPCRs were revealed to be particularly important in breast cancer (Lyu et al., 2021). Our observation further supports that G_i/o_- and G_q/11_-coupled GPCRs signals may play an important role in the cancer development and progression, thus targeting the shared signaling pathways may be beneficial to the treatment for PRAD. However, the preference is not obvious for the coupling of 46 DEGpcrs to G_i/o_ and G_q/11_. Of the 46 DEGpcrs, 7 are coupled to G_i/o_, five are coupled to G_q/11_, 6 are coupled to G_s_, 2 are coupled to both G_i/o_ and G_q/11_, and 26 have unknown G protein linkage. Certainly, these findings are only derived from the analysis of primary prostate tumor samples and limited information about G protein linkages, and thus more efforts are required to further reveal the roles of GPCRs coupling with different G proteins in the future.

### Somatic mutations of GPCRs in primary PRAD

**FIGURE 2:**
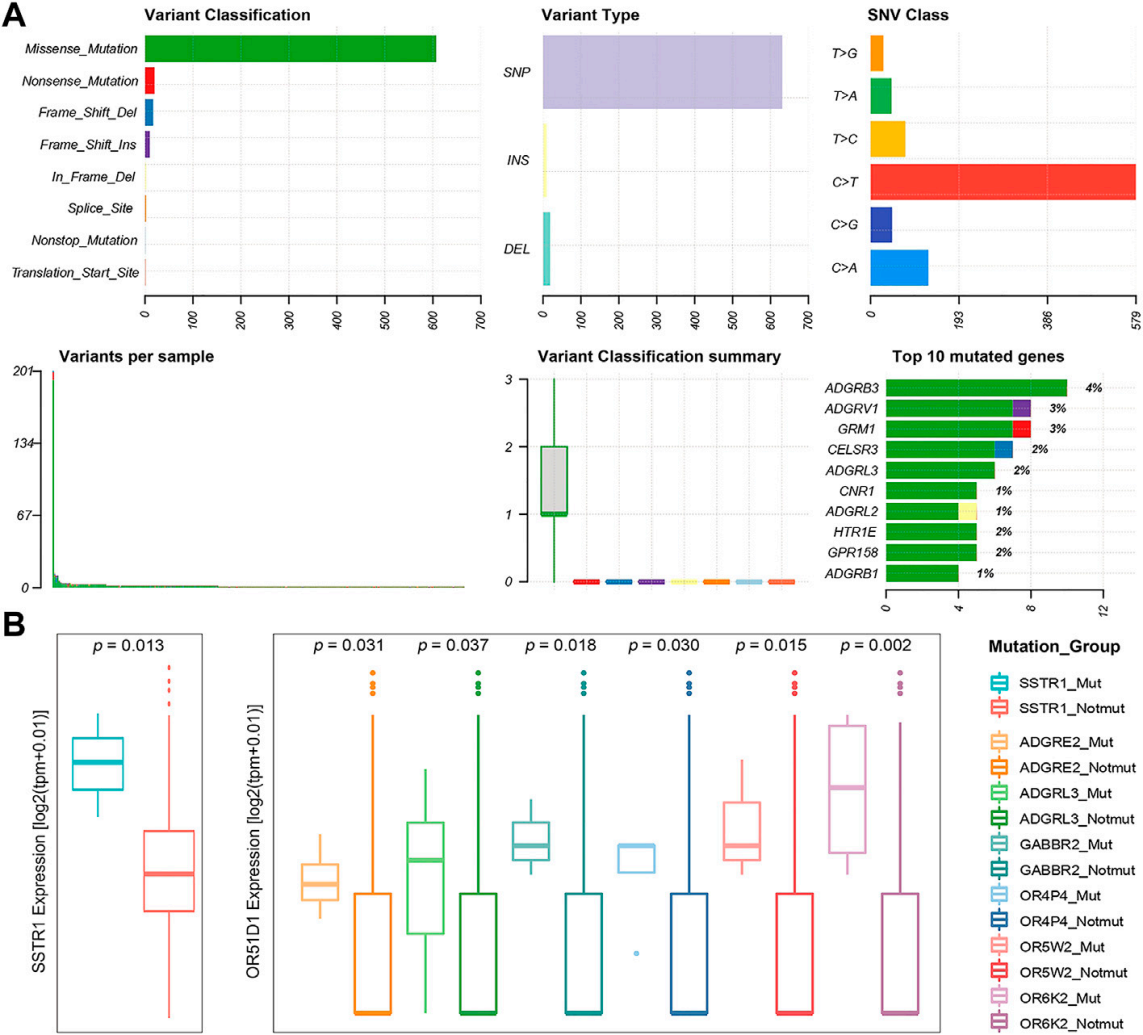
Statistics of GPCR Mutations. **(A)** A landscape of the GPCRs mutated in 276 primary prostate tumor samples generated by Maftools visualization module. **(B)** Boxplots of the expression levels of *SSTR1* (left) between the *SSTR1* mutated and unmutated groups, and *OR51D1* (right), an example of a gene that is dysregulated between the mutated and unmutated tumor samples of the other GPCR.

Many thousands of mutations occur during tumorigenesis, but only a few are able to confer selective growth advantage to cancer cells, which are critical to their tumorigenic capacity. Thus, identification of “cancer driver genes” is import to develop efficient cancer detection and therapeutic approaches (Stratton et al., 2009; Stratton Michael, 2011; Martínez-Jiménez et al., 2020). To this end, we first analyzed publicly available somatic variant calls in mutation annotation format (MAF) files of primary PRAD (n = 484) from the TCGA. It was found that approximately 57.02% of tumor samples (276 out of 484) present at least one GPCR mutation. Consequently, a total of 660 somatic mutations in GPCRs are identified, including single nucleotide variants (SNVs) and small insertions and deletions (InDels). Figure 2A shows the summary of the GPCRs mutated in the 276 primary PRAD tumor samples, in which missense mutations exhibit the highest proportion among all the mutation types. At base substitution level, transitions were found to be more common than transversions, with the C > T mutation occurring predominantly. However, GPCRs exhibit a pretty low mutation frequency, with only 2.39 mutations each sample on average. Figure 2A further shows the top 10 mutated GPCRs whose mutation frequencies are lower than 4%. The most frequently mutated *ADGRB3* occurs in only 3.62% (10/276) of the samples. These observations indicate that the low mutation frequency of GPCRs likely has contributed to the limited use of GPCR-targeted drugs as cancer therapeutics (Xu et al., 2000; Insel et al., 2018; Sriram et al., 2019).

Subsequently, we used the MutSigCV algorithm to identify 13 significantly mutated GPCRs (SMGpcrs, *p* value <0.05) in tumor samples (Lawrence et al., 2013). Supplementary Table S4 summarizes their names, *p* values of significance and mutation frequencies. As expected, the mutation frequency of these SMGpcrs is pretty low, consistent with a previous study on the identification of oncogenic drivers (Armenia et al., 2018), which indicated that the incidence of SMGs in prostate cancer follows a long-tailed distribution with many genes mutated in less than 3% of cases. However, these mutations still probably affect a large number of patients due to the high incidence of PRAD (Armenia et al., 2018). Therefore, we may conjecture that the GPCR mutations revealed are still useful for understanding the PRAD mechanisms, although they are unlikely to stand out on a genome-wide scale due to their low frequency. In fact, many SMGpcrs identified were reported to participate in various cancer-related processes. For example, multiple mutations in metabotropic glutamate receptor 1 (*GRM1*) gene have been implicated in various tumors including PRAD, which are associated with altered protein function, downstream pathways, migration, and angiogenesis, thus contributing to tumorigenic progression (Koochekpour et al., 2012; Esseltine et al., 2013; Ali et al., 2014; Shah et al., 2019). Abnormal expression of *CNR1* has been observed in prior studies and was found to be correlated with the severity and prognosis of tumors like hepatocellular carcinoma, ovarian cancer, and PRAD (Chung et al., 2009; Messalli et al., 2014; Mukhopadhyay et al., 2015; Liu et al., 2020), but there are conflicting publications regarding the role of the cannabinoid receptor in tumor proliferation (Hart et al., 2004; Pisanti et al., 2013), for example, some groups indicated that the cannabinoid receptor exerts antitumor effects (Ramer and Hinz, 2016; Wilkie et al., 2016) while others suggested its tumor-promoting effect (McKallip et al., 2005). In addition, 8 of the 13 SMGpcrs are the olfactory receptors (ORs). Unfortunately, little attention has been paid to other potential of this receptor family besides olfaction, thus there has been very limited information for functional roles of many ectopically expressed ORs. However, in the past decade, studies bloomed linking the ectopically expressed ORs to cancer initiation, development and progression (Chen et al., 2018; Maßberg and Hatt, 2018). Certainly, the GPCR’s mutation rates of <5% is only derived from the data of 484 primary prostate tumor samples, thus more tumor samples should be needed to further confirm the prevalence and functional role of GPCR mutations in the future. In addition, to gain insight into the impact of these mutations on the GPCR activity, we further examine whether they are clustered on the hot spots of the related GPCRs. Herein, we focused on the 13 significantly mutated GPCRs (SMGpcrs). It was observed that three mutations (S1169L, P1079S, R981C) occur at the C-terminus of GRM1, which is a member of the metabotropic glutamate receptor (mGluR) family. As revealed, mGluRs can dimerize and bind to a variety of downstream transducers while their intracellular C-termini domains are the main targets (Enz, 2007). Thereby, the intracellular C-terminus of mGluRs is critical for designing drugs that interfere with specific protein-protein interactions (Enz, 2012). P341L mutation of HTR1E occurs in the conserved NPxxY motif. As recorded in the Uniprot database (2021), the NPxxY motif plays important role in the ligand-induced GPCR conformation change and signaling. The M461V mutation was observed to occur at the C-term in CB1R while a recent report highlighted the importance of the CB1R C-terminal domain in polarized trafficking and surface expression in cultured neurons (Fletcher-Jones et al., 2019). Unfortunately, the GPCR database has no record for the other 10 significantly mutated GPCRs and we do not find information regarding their structures and hotspots from literature. Therefore, we cannot estimate whether these mutations will impact the activities or functions of GPCRs. However, the observations from the three significantly mutated GPCRs suggest that these mutations indeed play important roles in the GPCR function. We hope that these results will attract more attention to these understudied GPCRs in the future.

Wnt signaling is one of the key cascades regulating development and stemness, which is closely associated with cancer (Zhan et al., 2017). The high frequency of WNT pathway mutations in many different cancers underscores the importance of this signaling in carcinogenesis. Therefore, besides the frequency statistics on the cancer driver genes, we also focused on the GPCR mutations involving the WNT pathway in the primary PRAD. As shown in Supplementary Figure S2, the WNT pathway carries nine GPCRs with somatic mutations (seven FZD genes and two LGR genes), implicating their oncogenic-related functions.

To investigate the potential impact of the somatic mutations on the gene expression, we integrated the gene expression data and the mutation one from 481 primary PRAD patients, and selected 50 GPCRs that mutate in at least three samples. Nearly half (43.47%, 20 out of 46) of the DEGpcrs don’t harbor any mutation. Only *CNR1* is identified by MutSigCV to be a SMGpcr and is significantly downregulated in the tumor samples, implying its potential tumor suppressive effect. Using a Fisher’s exact test, we evaluated whether the expression of a specific GPCR is significantly higher in the mutated samples than those lacking mutations. The result shows that none of the 50 mutated GPCRs displays significant enrichment in the highly expressed groups. In addition, we divided the tumor samples into two groups according to the presence or absence of mutations of a specific GPCR gene, and performed differential expression analysis using a Wilcoxon test. Only *SSTR1* was found to be highly expressed in its mutated samples (Figure 2B). The above observations indicate that GPCR mutation is largely independent of their expression level and dysregulations.

Although the direct correlation between the mutation status of GPCRs and their mRNA expression is not significant, are their expressions associated with the mutation status of other genes? It is found that the mRNA expression levels of 39 DEGpcrs exhibit significant differences between the mutated and not mutated groups of the other genes. As shown in Figure 2B, *OR51D1* is significantly over-expressed in samples with mutations of *ADGRE2*, *ADGRL3*, *GABBR2*, *OR4P4*, *OR5W2*, and *OR6K2*. However, the correlation is not observed in its own mutant subgroups, as evidenced by Figure 2B and Supplementary Table S5. Based on all the aforementioned findings, it is reasonable to speculate that certain GPCRs are not significantly differentially expressed in PRAD tumors due to their own mutations, yet present to some extent correlations with somatic mutations in other GPCR genes. Certainly, the number of mutant samples involved in this study is limited, and additional large-scale studies are needed to validate these findings.

### Somatic copy number alterations of GPCRs in primary PRAD

**FIGURE 3:**
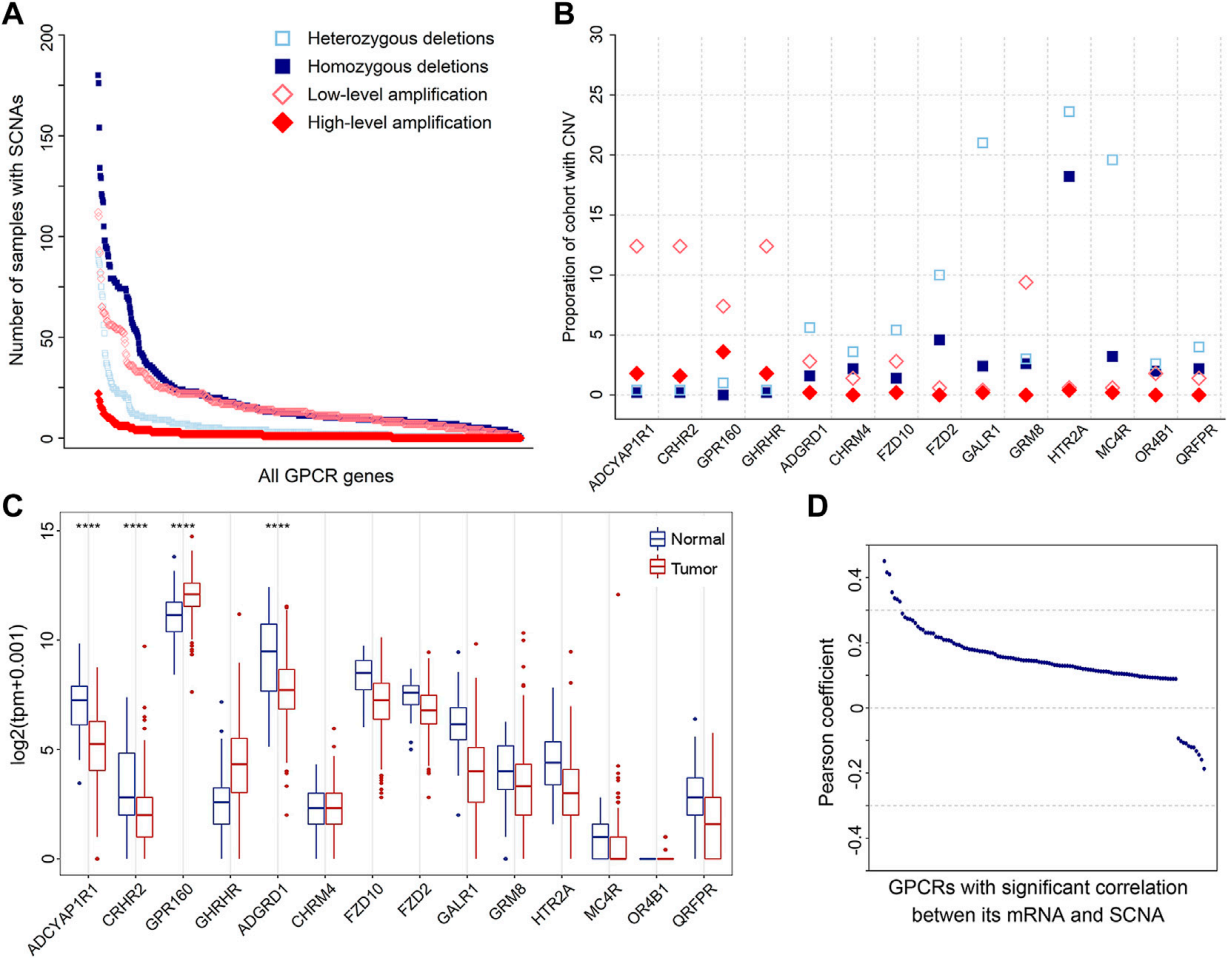
SCNAs of GPCRs in PRAD. **(A)** The number of heterozygous/homozygous deletions, and low-/high-level amplifications for all GPCRs. **(B)** Scatter plot of recurrent amplifications and deletions in 500 primary prostate tumor samples. **(C)** Box plots showing the expression of the significantly deleted and amplified GPCRs between the tumor and normal samples. **(D)** Distribution of Pearson’s correlation coefficients between the GPCR expression and its linear SCNA values.

SCNA is another molecular feature on the genomic level, which may cause the genome copy number of the affected cells to deviate from the normal diploid state such that affecting the stability of the genome and promoting the development of tumor cells (Albertson et al., 2003). Thus, we also analyzed SCNAs. The result shows that SCNAs occur extensively in primary prostate tumors. A total of 738 amplified and 634 deleted GPCRs were identified in 500 tumor samples, with a median of 27 amplifications (range 0–347) and 37 deletions (range 0–382) per tumor sample. The frequency distribution of samples with GPCR SCNAs is shown in Figure 3A. It can be seen that heterozygous SCNAs of GPCR genes are more common than homozygous SCNA events, which is consistent with a previous study on the GPCR SCNA events in pan-cancer (Sriram et al., 2019). Most GPCRs have a low frequency of SCNAs, as the SCNA of 571 GPCR genes occurs in 10% or less of tumor samples. However, there are still some GPCRs presenting frequent SCNAs, for example, the most frequently deleted *ADRA1A* and amplified *FZD6* occur in 52.20 and 26.80% of the tumor samples analyzed, respectively.

It was proposed that amplified and deleted GPCRs may have potential as biomarkers (Vang Nielsen et al., 2008; Sriram et al., 2019). Thus, we utilized GISTIC2.0 to detect significantly recurrent SCNA events of GPCRs (Mermel et al., 2011), and 52 significantly altered regions (*q* value <0.25) were identified. Figure 3B shows the identity and frequency of these significant GPCR SCNAs. The 23 amplified peak regions encompass 4 GPCRs on the chromosomes 3q and 17p, while recurrent arm-level amplifications also occur in the 2 chromosome arms. The 29 deleted peak regions harbor 10 GPCRs, three of which are on chromosomes 13q and 18p that show recurrent arm-level deletions. Therefore, the significant amplifications of *ADCYAP1R1*, *CRHR2*, *GPR160*, and *GHRHR*, and the deletions of *GALR1*, *MC4R*, and *HTR2A* are possibly attributed to their corresponding recurrent arm-level SCNAs.

By assessing the expression of GPCRs with the above recurrent focal SCNA events, we further explored the power of SCNAs in explaining why the expression levels of DEGpcrs are significantly dysregulated in cancers. The results reveal that among the four amplified GPCRs, only *GPR160* is over-expressed in primary prostate tumor samples, while the other two (*CRHR2* and *ADGYAP1R1*) are significantly under-expressed. Except for *ADGRD*, the expressions of the 9 deleted GPCRs don’t show statistically significant differences between the tumor and normal samples. These observations indicate that the expression of GPCRs could not be inferred from their copy number variant status. Except for *GPR160* and *ADGRD1*, SCNA alone does not generally predict the direction and extent of expressional dysregulation in prostate tumors compared to normal tissues (Figure 3C).

To further test the correlation between SCNA and mRNA expression of GPCRs, we extracted 491 primary PRAD patients with both mRNA and CNV data available and calculated their Pearson correlation coefficients. 611 of 736 GPCRs with both SCNA and mRNA data do not present significant correlations between the two features. Even though significant correlations were observed in the other 125 GPCRs, the associations are generally weak with most correlation coefficients below 0.3. Only 7 pairs exhibit correlation coefficients in the range of 0.3–0.5, as evidenced by Figure 3D. These observations indicate that no direct correlation exists between SCNA and mRNA expression for most GPCRs. Moreover, we further evaluated the GPCR mRNA expression between different PRAD groups classified by the SCNA status of the specific gene. Similarly, no significant differential expression was observed, further confirming that there is usually lack of the significant correlation between SCNA and mRNA expression of GPCRs.

### DNA methylation alterations of GPCRs in primary PRAD

**FIGURE 4:**
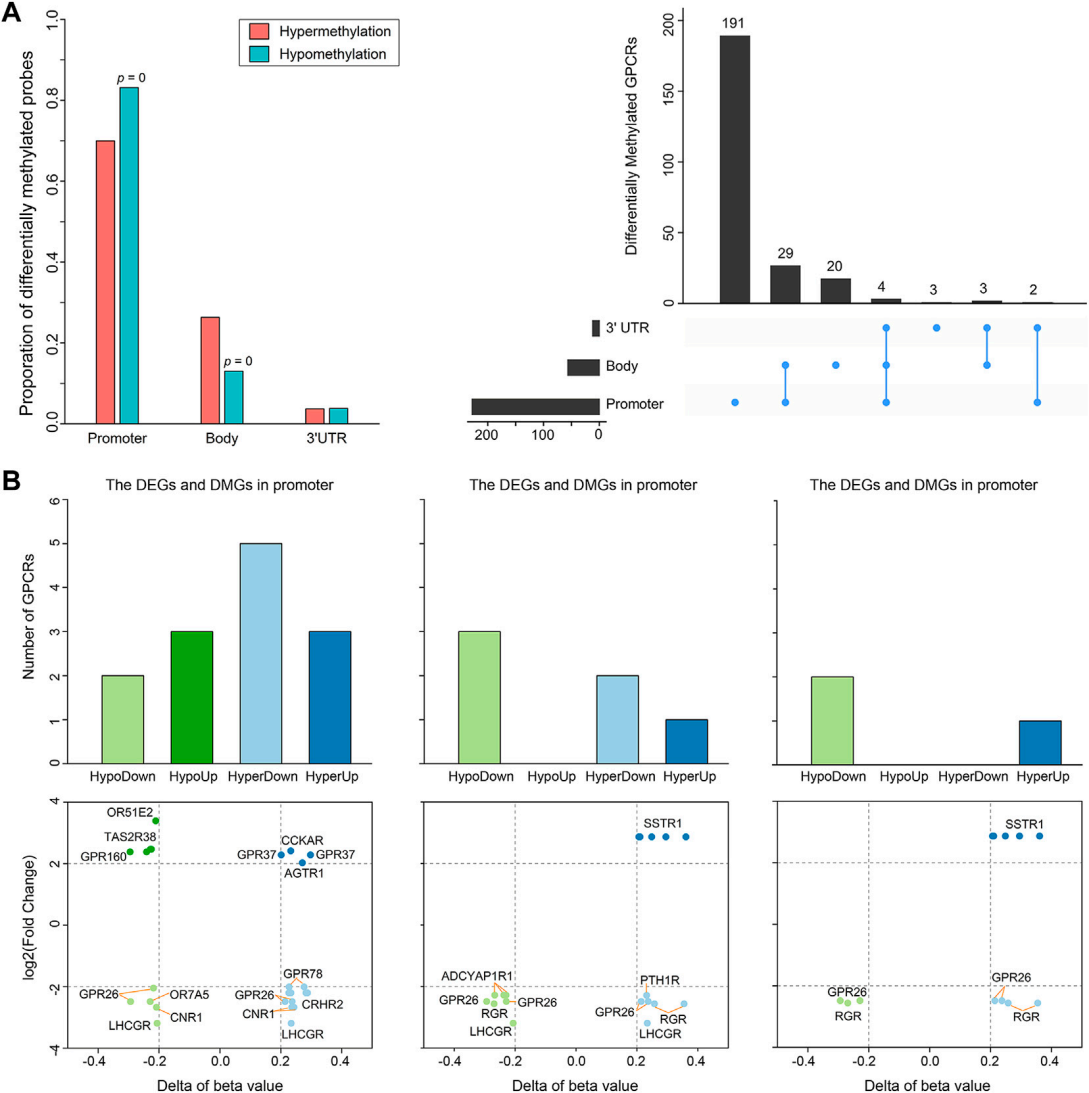
Differential methylation of GPCRs in the 491 PRAD patients. **(A)** Bar plot and Upset showing the number of DMGpcrs. **(B)** Bar plots and scatter plots showing the number and identity of DMEGs, respectively.

Besides somatic mutations and SCNAs, epigenetic changes also contribute to tumorigenesis (Villanueva et al., 2020). DNA methylation is the most widely studied epigenetic mechanism, and its alteration generally results in malignant tumors mainly by means of DNA hyper- or hypomethylation (Pan et al., 2018). Thus, we extracted DNA methylation data from 50 PRAD patients with matched control and tumor samples to conduct a comparative analysis. Statistically significant methylation changes (|Δβ| > 0.2 and adjusted *p*-value < 0.05) were observed in 504 regions of 252 GPCR genes, in which 243 differentially methylated positions (DMPs) are hypermethylated and 261 DMPs are hypomethylated in the primary prostate tumor samples, as reflected by Supplementary Table S6. Considering the fact that there are different methylation characteristics and functions among different genomic regions, we divided these 504 DMPs into 170 hypermethylated DMPs and 217 hypomethylated DMPs in the promoter region, 64 hypermethylated DMPs and 34 hypomethylated DMPs in the gene body, and 9 hypermethylated DMPs and 10 hypomethylated DMPs in the 3′UTR (Figure 4A, left). As shown in Figure 4A, 214 of the 252 significantly differentially methylated GPCRs (DMGpcrs) only appear in one region, 34 genes in at least 2 regions, and only 4 genes (*GPR26*, *GPRC5C*, *GRM1* and *OPRM1*) in all the 3 regions. The observations imply that the DNA methylation is region-specific in PRAD and certain genomic regions may be more susceptible to changes than others, for example, the promoter region that involves in the most frequent methylation changes. In fact, the promoter region has been a focus of attention in DNA methylation studies and its methylation level is considered to be closely related with cancer development (Saghafinia et al., 2018).

It is well known that DNA methylation can control gene expression without incurring any change to the genomic sequence; epigenetic changes could inappropriately cause transcriptional dysregulation, causing various diseases, including cancer (Anastasiadi et al., 2018). We hence explored the relationship between DNA methylation and mRNA expression of GPCRs. It has been reported that treating methylation sites and their located genes as single units may minimize noise from unrelated methylations and gene expression (Guo et al., 2019). Therefore, we preliminarily explored the relationship between dysregulated expression and aberrant methylation of GPCRs in different regions by coupling the DEGpcrs and DMGpcrs as single units. As a result, a total of 16 differentially methylated and expressed GPCRs (DMEGpcrs) were identified, which fall into 4 classes: HypoDown, HypoUp, HyperDown and HyperUp (Supplementary Table S7). HyperDown is the most common within promoter regions (5/13, Figure 4B), suggesting that this region causes gene silencing mainly through abnormal methylation, and oncogenes and therapeutic markers have established based on such association (e.g., CDKN2A (The Cancer Genome Atlas Research Network, 2012) and BRCA1 (Cancer Genome Atlas Research Network, 2011)); the Gene body region is dominated by HypoDown (3/6, Figure 4B), while only HypoDown and HyperUp are present in the 3′UTR region (Figure 4B), indicating that the aberration of DNA methylation and expression in the gene body and 3′UTR regions usually exhibit a consistent direction. The analysis above revealed the relationship between aberrant DNA methylation and expression preliminary.

To have a more accurate picture of the association between gene expression and DNA methylation, we conducted a correlation analysis on GPCRs from 494 primary PRAD patients having both two data types. Gene expression is often negatively associated with DNA methylation within promoter regions, but positively associated with DNA methylation in gene bodies. Specifically, among the 19 mRNA - methylation pairs in the promoter regions, 14 pairs show significantly negative correlation with Pearson r < 0, while in the gene bodies, 7/10 significant correlated pairs present Pearson r > 0. For example, the expression of *GPR26* is negatively correlated with the methylation level of cg04549162 and cg11893763 (Pearson r < −0.15), which locate around 200 kb upstream of *GPR26*. In contrast, the expression of *GPR26* presents positive correlation with the methylation of cg25912428 (Pearson r = 0.2359) locating in the gene body. The observation indicates that the overexpression of *GPR26* in primary prostate tumors is probably due to its hypermethylation in promoter and hypomethylation in gene body. In fact, the positive and negative correlations have been widely reported, i.e., hypermethylation of CpG sites in promoters typically leads to transcriptional silencing, whereas hypomethylation of CpG sites in a gene body frequently results in an increase in gene expression (Shen and Laird, 2013; Sun et al., 2018). In addition, we identified a new regulatory region, the 3′UTR, with a significant positive correlation between the level methylation and expression, such as *GPR26* - cg13557752, *RGR* - cg14856914, *SSTR1* - cg04265797, and *SSTR1* - cg04573550 (Supplementary Figure S3). All the observations indicate that the alteration in expression might be due to the degree of DNA methylation, and the correlation between them is highly relied on where the DNA methylation occurs: the abnormal decrease of GPCR mRNA expression in primary PRAD is likely a result of hypermethylation in promoters and hypomethylation in gene body and 3′UTR regions. In summary, our correlation analysis provides insights into regulatory relationships between DNA methylation and expression of GPCRs in PRAD.

### Identification of GPCRs as potential therapeutic targets for drug repurposing

Characterizing the genome, epigenome, transcriptome and their interactions is vital for our understanding of cancer behavior, not only for deepening insights into cancer-related processes but also for future disease treatment and drug development. Based on the above multi-omics analysis, we identified significantly altered GPCR members in primary PRAD. Drug development targeting such receptors should be helpful for the development of effective anticancer therapies. To this end, we selected *GPR160* and *CRHR2* which significantly altered at the multi-omics layers as representatives to conduct structure-based virtual screening, which is a powerful and widely used computational approach for the identification of lead compounds (Zhao et al., 2021).

**FIGURE 5:**
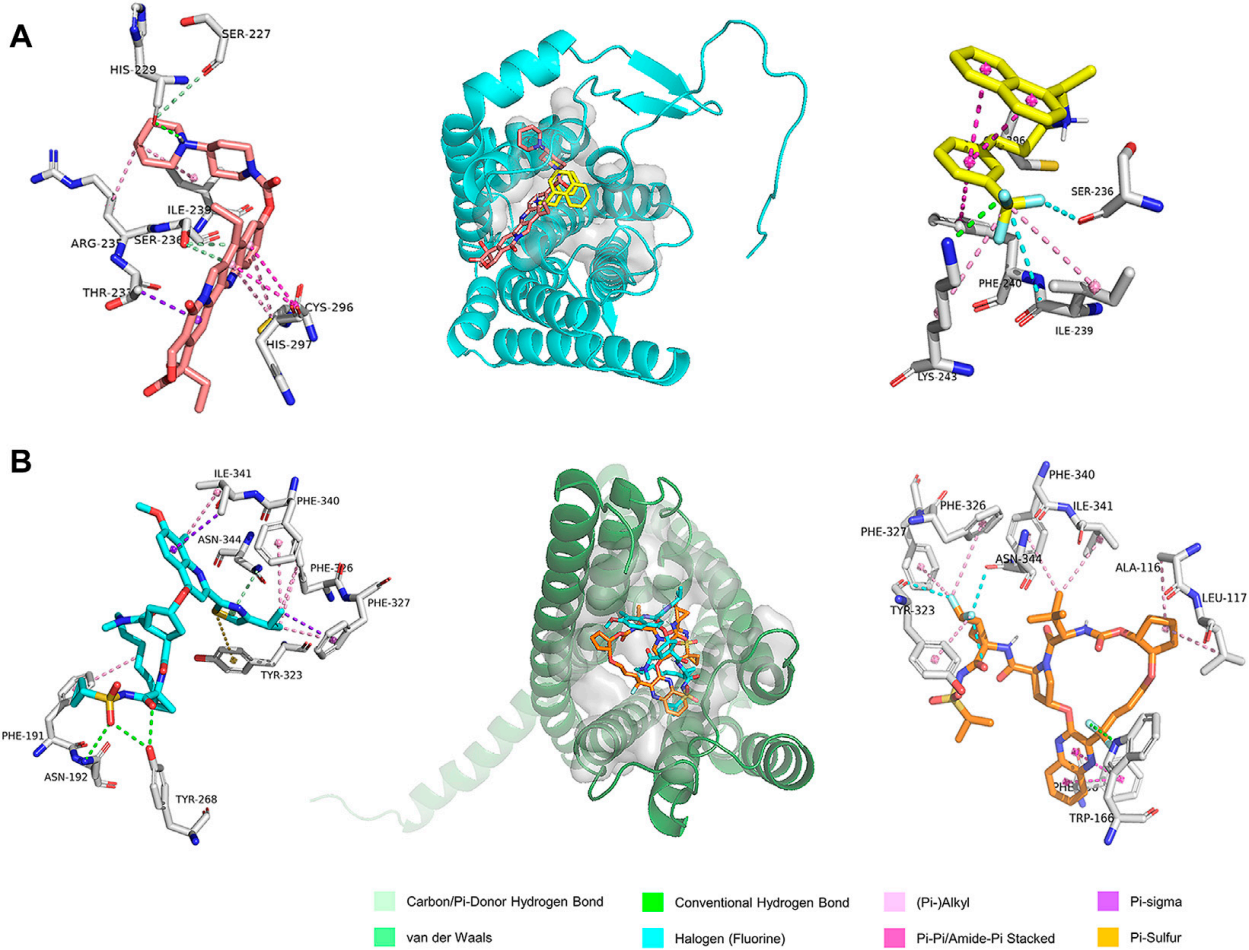
The predicted binding modes of the ligands with **(A)** GPR160 and **(B)** CRHR2 at the ligand binding pocket (dashed box). Different ligands are represented by different colored sticks, salmon: Irinotecan, yellow: Cinacalcet, orange: Simeprevir, and cyan: Glecaprevir.

GPR160 belongs to the class A GPCR subfamily and was de-orphanized recently (Yosten et al., 2020). As outlined above, GPR160 is significantly amplified and hypermethylated in the promoter region (TSS1500 and 5′UTR), along with upregulated gene expression. Therefore, GPR160 may be a promising drug target for the treatment of PRAD. Previous experimental studies have fully revealed the involvement of GPR160 in PRAD, including its expression dysregulation at both mRNA and protein levels, and the authors further demonstrated that the knockdown of GPR160 resulted in cancer cell apoptosis and growth arrest (Zhou et al., 2016; Guo et al., 2021). Therefore, GPR160 may be a promising drug target for the treatment of PRAD. However, there has not been available crystal structure for GPR160. Thus, we used the GPR160 structure (Q9UJ42) predicted by AlphaFold at http://alphafold. Ebi. ac.uk (Varadi et al., 2021). As known, AlphaFold is a deep learning-based approach recently developed, showing remarkable success in predicting the protein structure. Then, Fpocket algorithm [86] was used to identify its ligand binding pockets, leading to ten pockets. The top scoring pocket consisting of 22 residues was selected for the subsequent virtual screening to 1615 FDA-approved drugs from ZINC (Irwin and Shoichet, 2005), which is a free database of purchasable compounds for ligand discovery and virtual screening. To obtain the stable protein-ligand complex structure, we further used 100 ns MD simulation on the top eight hits (Supplementary Table S8) of the docking result. As evidenced by RMSDs of GPR160 in Supplementary Figure S4A, all the eight systems achieve equilibriums. To obtain reliable evaluation on the binding affinity between GPR160 and the eight ligands screened, MM-GBSA was used to calculate the ligand-receptor binding free energy for the eight systems and the result is listed in Supplementary Table S8. Despite the strongest affinity of Trypan Blue to GPR160, it is not further considered as a candidate in this study due to the fact that Trypan Blue generally acts as stain and has no any reports involving the disease treatments. Here, we focused on Cinacalcet (−23.68 kcal/mol) and Irinotecan (−20.43 kcal/mol), which also show strong binding affinities to GPR160 (vide Table 1). As shown in Figure 5A, the two ligands present diverse interactions with GPR160, for example, the binding of Cinacalcet to GPR160 is mainly attributed to hydrogen bonding with SER236, LYS243 and CYS296, π-π bonding with PHE240, and halogen bonding with ILE239. The tight binding of Irinotecan to GPR160 is due to hydrogen bonding with HIS229 and SER236, π-σ bonding with THR233, amide-π interacting with CYS296, and π-alkyl bonding with ILE239. Additionally, existing studies suggested the anticancer potential of the two drugs. Cinacalcet, which is approved by FDA to treat secondary hyperparathyroidism (Nemeth et al., 1998), has shown therapeutic application for hepatocellular carcinoma (Zheng et al., 2021), and has been reported to reduces neuroblastoma tumor growth in preclinical models (Masvidal et al., 2017). Irinotecan as a camptothecin-derived drug that is the first approval for cancer treatment has contributed to the treatment of multiple cancers worldwide, including advanced colon cancer, non-small cell lung cancer, and cervical cancer (Hsiang and Liu, 1988; Bailly, 2019). The existing reports support that Cinacalcet and Irinotecan may be promising candidates for the treatment of PRAD, also confirming the reliability of our results from the virtual screening and the molecular dynamics simulation.

**TABLE 1:** Binding energies, derived from MM/GBSA calculation**,** of the top two compounds selected in virtual screening for *GPR160* and *CRHR2* respectively.

ZINC ID	Drug name	Weight^a^	Binding energy^b^
ZINC000001550499	Cinacalcet	357.41	−23.68 ± 2.69
ZINC000001612996	Irinotecan	586.69	−20.43 ± 3.24
ZINC000164528615	Glecaprevir	838.88	−47.39 ± 4.74
ZINC000164760756	Simeprevir | Olysio	749.96	−44.87 ± 3.25

a g/mol.

b Kcal/mol.

The analysis above already indicates that CRHR2 is significantly amplified, hypermethylated in the promoter region (TSS200 and TSS1500) and under-expressed. CRHR2, belonging to class B1 GPCRs, is best known as regulators of the stress response in the central nervous system. Although there have been seldomly reports about CRHR2 in cancer, its role in tumor formation and angiogenesis is becoming increasingly studied. For example, low or absent CRHR2 expression was found in exocrine ductal pancreatic carcinomas, PRAD and non-small cell lung cancer, in line with our findings. It was reported that expression loss of CRHR2 may contribute to prostate tumorigenesis, progression and neoangiogenesis (Reubi et al., 2003; Tezval et al., 2009). In addition, recent studies indicated that hypermethylation of CRHR2 may be responsible for lowered tissue expression of this protein (Kasprzak and Adamek, 2020). Overall, CRHR2 has been found to be dysregulated in expression and methylation in multiple cancers, and involved in angiogenesis and tumor progression, implying that it may be served as potential therapeutic targets in cancer. Thus, we selected the cryo-EM structure of CRNR2 co-crystallized with Urocortin 1 from human (PDB Identifier: 6PBI) (Bentley et al., 2020), and select its amino acid residues within 4 Å of the ligand binding site in 6PBI as the binding pocket for virtual screening. Similarly, we conducted 100-ns MD simulation on 8 complex systems with the top hits in the docking score and calculate their MM/GBSA energies. Glecaprevir (−47.39 kcal/mol) and Simeprevir (−44.87 kcal/mol) present the strongest binding affinity (vide Table 1), implying their potential as promising drugs to CRHR2. Figure 5B shows the predicted binding modes for the two drugs with CRHR2. Similarly, the strong binding affinities are also attributed to the diverse interactions between the two drugs and CRHR2, as reflected by Figure 5B. Glecaprevir, which has been used as the hepatitis C virus (HCV) NS3/4A protease inhibitor (Lamb, 2017), is proposed to have antitumor potential for the first time in our work report. Simeprevir, originally served as a hepatitis C antiviral agent, was recently repurposed as an effective anti-cancer agent that simultaneously inhibits two important pathways known to be involved in both tumorigenesis and treatment resistance (Park et al., 2017; Kattan et al., 2021). These previous observations also to some extent support our predictions.

However, it is noted that we screened the four high-affinity drugs for the potential targets (GPR160 and CRHR2) and evaluated the anticancer potential only by means of the computational way. As known, the docking and structure-based virtual screening aim to predict the binding mode of a ligand and its affinity to the target protein, but cannot distinguish its efficacy like agonists or antagonists or inverse agonists (Ballante et al., 2021), which need further experimental evaluation like functional assays. In addition, our screening to the potential ligands is based on the classic pharmacological dogma “one drug-one target”. Although the dogma has been dominant in drug discovery for decades, it has been recognized that inhibition of a single target is often not sufficient to generate optimal therapeutic benefit for the disease that displays polygenicity (e.g., cancer, psychiatric diseases) or involves complex biological signaling networks and feedback loops (Palve et al., 2021). In the case, multitargeted drugs and drug combinations may represent valuable complements, which are emerging as new paradigms in drug discovery (Anighoro et al., 2014; Palve et al., 2021).

### Prognostic value of GPCRs in primary PRAD

**FIGURE 6:**
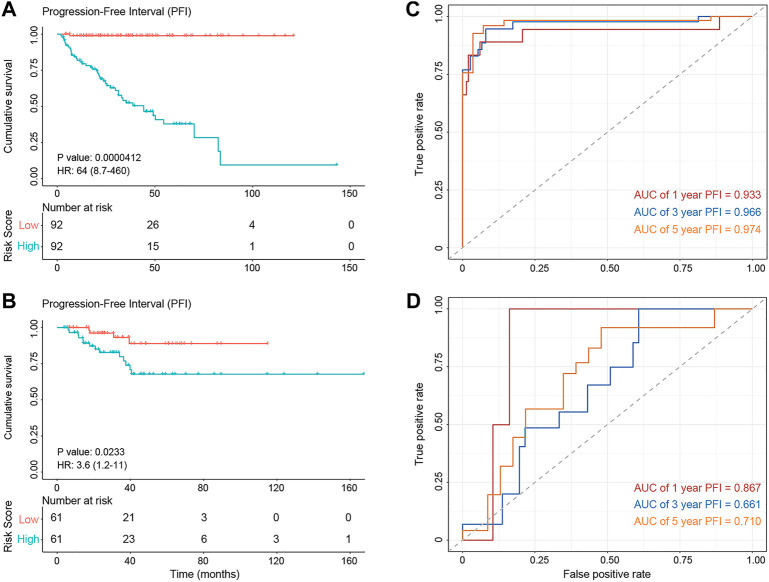
The diagnostic performance of the prognostic model. **(A,B)** Kaplan–Meier survival analysis showing PFI differences between the high-risk (red) and low-risk (blue) groups in the training set **(A)** and test set **(B)**. **(C,D)** The ROC curve showing the AUC value of the risk model in the training set **(C)** and testing set **(D)**.

GPCRs that exhibit abnormalities at the multi-omics layers should provide clues for the prognosis development, besides their value in the drug discovery. Thus, we further investigated the correlation between the abnormal molecular characteristics of GPCRs and the prognosis of PRAD patients, through which GPCR molecular features significantly associated with prognosis can be identified on one side and we also hope to build an accurate prognostic model on the other side. As some clinical features like age and tumor stage were reported to influence the prognosis of patients as well (Li et al., 2021), they were taken into account to construct the prognostic model. Supplementary Table S9 shows available clinical features collected. The univariable analysis reveals that some clinical features, gene expression and SCNA status of some GPCRs are significantly associated with PFI (*p* < 0.05). However, none of the GPCR methylation and somatic mutations reaches the statistical significance, which is different from the observations in some previous survival analyses on other genes and cancers. For example, *EGFR* and *TP53* mutations are accepted as prognostic factors in advanced non-small cell lung cancer (Jiao et al., 2018). *MUC16* mutations were found to be associated with improved outcome in patients with gastric cancer (Zeng et al., 2020). Several biomarkers based on DNA methylation changes have been identified in colorectal cancer (Gutierrez et al., 2021). In order to reduce the influence of collinearity among genes in identifying the prognostic predictors and to build a more accurate prediction model, we further performed a stepwise multivariate Cox regression analysis on the training set (n = 184) by including features significantly associated with prognosis derived from the above univariate analysis. Finally, the prognostic model is obtained by using 3 clinical features, mRNA expression of 19 GPCRs, and SCNA of 7 GPCRs, in which 13 features with HR < 1 are considered as protection factors, and 16 features with HR > 1 are risk factors. Supplementary Figure S5 lists the 29 predictors in detail. On the basis of the risk prognostic model, the risk score of each sample can be obtained. Kaplan-Meier curve in Figure 6 shows that the samples in the high-risk group exhibit worse PFI than those in the low-risk one, indicating the prognostic signature of risk score is effective (training set: log rank *p* = 4.12e-5, HR = 64; test set: log rank *p* = 0.023, HR = 3.6). In order to compare the sensitivity and specificity of the risk score on the prognosis of patients with primary PRAD, time-dependent receiver operating characteristics (ROC) analysis was also performed, and the areas under the ROC curves (AUC) in the training set at 1, 3, and 5 years are 0.974, 0.966, and 0.933, respectively (Figure 6C). For the testing set, they are 0.87, 0.66, and 0.71, respectively (Figure 6D). Collectively, the 29 features involving the abnormal mRNA expression and copy number variants of GPCRs may serve as potential biomarkers to predict primary PRAD prognosis. Combined with the observations from the omics analysis above, it suggests that the features significantly associated with disease at the single-omics level may not serve as effective prognostic markers. For example, some GPCRs mutated significantly are not associated with the prognosis, possibly due to the fact that cancer is a disease involving multi-omics dysregulations like genetic alterations, differential DNA methylations and transcriptomic disorders. Thus, the features found at a single omic layer may be altered by subsequent regulation or modification. Thus, the combination of the multi-omics data leads to more accurate predictions, suggesting that survival prediction in oncology would likely benefit from multi-omics analysis.

## Conclusion

In the work, we offer the first comprehensive landscape of multi-omics features of the GPCR family in the primary PRAD using an unbiased (-ome) approach. Several important observations are obtained:

1) GPCRs exhibit low expression levels and mutation frequencies, which should contribute to less focus on GPCRs in oncology. However, the mRNA expression and DNA methylation analysis identify 46 and 255 disease-related GPCRs, respectively, complementing information lack in the genome analysis.
2) The associations between distinct omics layers are found to be different. Most receptors don’t exhibit a significant correlation between the genome and transcriptome while a tight association is observed between the transcriptome and epigenome of GPCRs, confirming the complex regulatory mechanism from DNA to RNA.
3) Four drugs (Cinacalcet, Irinotecan, Glecaprevir, and Simeprevir) targeting GPR 160 and CRHR2, which show significant alterations at different levels, are identified as potential candidates to reposition for prostate cancer by the virtual screening and molecular dynamic simulations.
4) The combination of 3 clinical characteristics and 26 GPCR molecular features identified by the transcriptome and genome exhibit good performance in predicting progression-free survival in patients with the primary PRAD, thus providing new potential biomarkers for the clinical decision.

In a whole, these observations on the GPCR family at the genomic, epigenomic, and transcriptomic levels provide new insights for understanding the mechanism of the primary PRAD, theoretically revealing the therapeutic and prognostic potential of GPCRs in PRAD. In addition, our result further confirms that the analysis of just one omics level generally provides a biased and incomplete snapshot in the complex disease progression, most probably missing some key cancer drivers. Thus, the integrated analysis considering multi-omics is beneficial to the development of new therapeutic strategies and prognostic markers. However, it is noted that our data from the public databases (TCGA and GTEX) were generated from whole blood or specific tissue samples, thus cannot capture the complex heterogeneity of single cells or the regulatory relationships between them. Recently, single-cell technologies are advancing. It is likely to generate omic data on single cells from different tissue types of interest in the future such that can accelerate new and more refined analyses.

## Materials and methods

### Data collection

Integrating data from the IUPHAR/BPS Guide to PHARMACOLOGY (GtoPdb, www.guidetopharmacology.org) (Alexander et al., 2019) and previous reports (Alexander et al., 2011;; Maiga et al., 2016, we compiled an annotation file of 766 GPCRs, including endogenous GPCRs (response to endogenous agonists), taste andolfactory members. We then manually check this list in NCBI Gene database, and full details of the receptor family are provided in Supplementary Table S1.

To comprehensively characterize the GPCR family in patients with primary PRAD, we collected genomic, transcriptomic and epigenomic data from publicly available databases, including The Cancer Genome Atlas (TCGA) and the Genotype-Tissue Expression (GTEx). Specifically, we downloaded both the mutation annotation format (MAF) files and SCNA data from GDC Data Portal (RRID:SCR_014514, https://portal.gdc.cancer.gov/). The RNA expression data generated by TCGA (Chang et al., 2013) and GTEx (Lonsdale et al., 2013) was obtained from the UCSC Toil RNA-seq recompute data hub (accessed on 13 December 2020) (Vivian et al., 2017) and the DNA methylation profiles came from the TCGA Hub—PRAD, both of which are stored at UCSC Genome Browser (RRID:SCR_005780, https://genome.ucsc.edu) (Goldman et al., 2020). In addition, the clinical information was retrieved from TCGA, UCSC Xena and Broad GDAC Firehose (https://gdac.broadinstitute.org/), which is listed in Supplementary Table S9.

### mRNA expression analysis

Firstly, we applied the “DESeq2” package with the threshold of |log_2_ fold-change| > 2 and adjusted *p*-value < 0.05 to select DEGpcrs in the primary prostate tumor samples compared with non-tumor ones (Love et al., 2014). Then, the class enrichment analysis was carried out for the over-or under-expressed group, using the R *fisher. test* function. Finally, the “corrplot” package was used to calculate Pearson correlation of the tumor gene expression between DEGpcrs and other protein-coding genes. Then, genes with a *p* value <0.05 were identified to perform enrichment analysis by the Metascape web-based portal (http://metascape.org) (Zhou et al., 2019).

### Somatic mutation analysis

Maftools (Mayakonda et al., 2018), which is already implemented as a R package, was applied to annotate, analyze and visualize the GPCR mutations from the MAF file of primary PRAD. The definition of SMGpcrs in tumor samples was performed by running the MutSigCV software with default parameters (Lawrence et al., 2013). Genes with *p* < 0.05 were considered to be significantly mutated.

### Somatic copy number alteration analysis

SCNAs of primary PRAD patients were analyzed by the GISTIC2 algorithm (Mermel et al., 2011), which is freely available as a module on the GenePattern web server at https://cloud.genepattern.org/gp/pages/index.jsf. The parameters were set as follows: “-refgene: Human_Hg38.UCSC.add_miR.160920. refgene.mat, -ta (-td): 0.25, focal length cutoff: 0.70, -genegistic: yes, -conf: 0.9, -qvt: 0.25, -broad: yes, -armpeel: yes”.

### DNA methylation analysis

Differences in DNA methylation levels between the primary prostate tumor samples and the non-tumor ones were quantified using the *ChAMP* package (RRID:SCR_012891) (Morris et al., 2014). We determined DMPs with a threshold of |delta β-value| > 0.2 and adjusted *p*-value < 0.05. DMPs were subsequently classified into different regions based on annotation from *ChAMP*: TSS1500, TSS200, Body, 1stExon, 3′UTR, and 5′UTR.

### Drug screening

There has been lack of a crystal structure for GPR160. BLASTP search and alignment did not identify a template with high sequence similarity. Thus, the predicted structure Q9UJ42 by AlphaFold was used for subsequent docking analysis (Varadi et al., 2021). Then, the potential binding region of GPR160 is identified by using Fpocket (Le Guilloux et al., 2009) that is a well-known pocket detection package based on the alpha sphere theory. As for CRHR2, the cryoelectronic microscopy structure of UCN1-bound CRF2R with the stimulatory G protein was obtained from GPCRdb (Kooistra et al., 2021) (https://gpcrdb.org/structure/refined/6PB1), and amino acids located at the vicinity of 4 Å from the ligand are considered as main binding residues. Thereafter, crystallographic ligands, stimulatory G protein and water molecules were excluded from the crystal structures. Polar hydrogens were then added to each protein by using Autodock tools (Morris et al., 2009). To achieve the goal of drug repurposing, a library of 1615 FDA-approved drugs obtained from the ZINC database, which is a free database of purchasable compounds for ligand discovery and virtual screening, were used for screening ligands (Irwin and Shoichet, 2005). Prior to docking studies, the proteins and small molecules were all saved into pdbqt format in preparation.

The virtual screening tasks were carried out by using AutoDock Vina, a freely available structure-based virtual screening docking program (Trott and Olson, 2010) with “exhaustiveness = 20, energy_range = 10, num_modes = 100” and other parameters being set to default. Based on the docking score, we selected the top eight hits of each receptor for molecular dynamics (MD) simulation. Membrane systems were constructed using the CHARMM-GUI Membrane Builder (Jo et al., 2009). Each system was simulated for 100 ns using AMBER16 (Case et al., 2016). In addition, we used molecular mechanics/generalized Born surface area (MM/GBSA) implemented in Amber16 to calculate the binding free energy of each complex, based on the last 20 ns equilibrium trajectory (Kollman et al., 2000).

### Survival analysis

In order to assess the prognostic value of each variable in PRAD, the univariate Cox analysis was adopted, in which the molecular and clinical characteristics were considered. The molecular characteristics involve important features at the somatic mutation and SCNA, DNA methylation levels and mRNA expression of GPCRs. To develop a prognostic model and evaluate its performance, we divided the cohort into a training set (60% of samples, n = 184) and a test set (40%, n = 122), in which the samples were proportionally allocated from each PFI type without replacement. Then, the significant features with *p*-value < 0.05 in the univariate Cox analysis were collected to perform a stepwise multiple Cox regression analysis on the training set. A patient’s risk score for PFI can be obtained by a linear combination of the regression coefficient derived from the multivariate analysis and the value of each significant variable, through which we could stratify patients into “high-risk” and “low-risk” groups. The PFI distribution of each group was described by the Kaplan-Meier curves and statistical significance was calculated using the log rank test. The predictive performance of the prognostic model was evaluated by c-index and ROC curves. Survival analysis and corresponding visualization were performed by using the R package “survival”, “survminer”, and “timeROC” (Blanche et al., 2013).

### Statistical analysis

For the correlation analyses between the gene expression and other molecular profiles, the following methods were used: 1) for continuous variables, including relative linear copy number values and DNA methylation levels, Pearson correlation was performed; 2) for categorical variables, the samples were divided into two groups based on a specific attribute (e.g., whether the specific GPCR was mutated), and then the non-parametric Mann-Whitney *U* test was performed to test the significant difference. *p* < 0.05 was considered statistically significant.

## Data Availability

The datasets presented in this study can be found in online repositories. The names of the repository/repositories and accession number(s) can be found in the article/Supplementary Material.
